# Roles for Countercharge in the Voltage Sensor Domain of Ion Channels

**DOI:** 10.3389/fphar.2020.00160

**Published:** 2020-02-28

**Authors:** James R. Groome, Landon Bayless-Edwards

**Affiliations:** ^1^ Department of Biological Sciences, Idaho State University, Pocatello, ID, United States; ^2^ Oregon Health and Sciences University School of Medicine, Portland, OR, United States

**Keywords:** countercharge, crystallography, electrostatic, ion channel, molecular dynamics, channelopathy, sliding helix model, voltage sensor domain

## Abstract

Voltage-gated ion channels share a common structure typified by peripheral, voltage sensor domains. Their S4 segments respond to alteration in membrane potential with translocation coupled to ion permeation through a central pore domain. The mechanisms of gating in these channels have been intensely studied using pioneering methods such as measurement of charge displacement across a membrane, sequencing of genes coding for voltage-gated ion channels, and the development of all-atom molecular dynamics simulations using structural information from prokaryotic and eukaryotic channel proteins. One aspect of this work has been the description of the role of conserved negative countercharges in S1, S2, and S3 transmembrane segments to promote sequential salt-bridge formation with positively charged residues in S4 segments. These interactions facilitate S4 translocation through the lipid bilayer. In this review, we describe functional and computational work investigating the role of these countercharges in S4 translocation, voltage sensor domain hydration, and in diseases resulting from countercharge mutations.

## Voltage-Gated Ion Channels and Electrical Excitability

The seminal work of Hodgkin and Huxley (1952) described voltage-dependent gating particles that determine membrane permeability to sodium and potassium ions during an action potential. Their experimental work culminated in a mathematical description of the action potential based on activation gates for sodium (m) and potassium (n) ions, and an inactivation gate for sodium (h). This paradigm describing the ionic basis for the action potential has since been upheld and augmented with biophysical explanations of the molecular phenomena through which voltage-gated ion channels (VGICs) dictate membrane excitability.

The description of the structure of DNA and the discovery of the genetic code allowed molecular biology to become an integral part of research into VGIC function. Genes for ion channels and other proteins involved in electrical signaling were cloned using strategies of peptide purification from a diversity of electrically excitable tissues [reviewed by (Dolphin, 2018)], and by employing unique phenotypes of *Drosophila* mutants to generate probes for gene cloning [i.e., *Shaker* potassium channel (Papazian et al., 1987)], and *para* sodium channel [reviewed by (Papazian et al., 1988; Ganetzky, 2000)].

**Figure 1:**
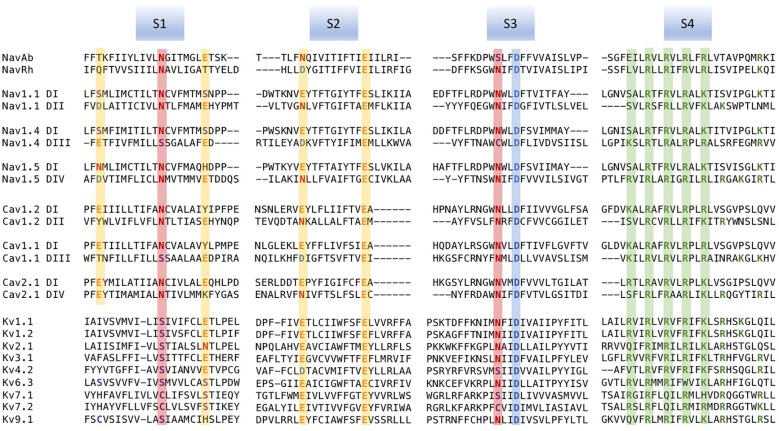
Multiple sequence alignments of voltage sensor domains (VSDs) (S1–S4) from a sample of voltage-gated ion channel (VGIC) alpha subunit domains. Prokaryotic sodium channels are Na_V_Ab from *Arcobacter butzleri* (Payandeh et al., 2011), and the NaChBac orthologue Na_V_Rh from *alpha proteobacterium* (Zhang et al., 2012). The remaining sequences are human. For each alignment, putative consensus negative charge regions are highlighted with boxes colored according to frequently observed residues (glutamate, yellow; asparagine, red; aspartate, blue). The first five positively charged residues in S4 are shown in green.

A striking pattern emerged in the amino acid sequences predicted from gene sequences for VGICs. Regularly spaced positively charged amino acid residues were observed within the fourth segment (S4) of hydrophobicity (Noda et al., 1984; **Figure 1**). Subsequent models of channel structure that were put forth included postulates regarding the interaction of positive and negative charges, and a helical screw mechanism of S4 translocation in channel activation (Greenblatt et al., 1985; Guy and Seetharamulu, 1986). With functional reconstitution of ion channel proteins in mammalian cells or *Xenopus* oocytes (Noda et al., 1986; Stuhmer et al., 1988; Timpe et al., 1988; Leonard et al., 1989), investigations of the structure to function relationships in VGICs focused first on the role of S4 segments in channel activation and fast inactivation (Stuhmer et al., 1989; Zagotta et al., 1989; Papazian et al., 1991). Progress in our understanding of the mechanisms of voltage-gating in ion channels has since been facilitated with advanced electrophysiological techniques, crystallography, and computational work. In this review, we examine the research efforts that describe the contributions made by negative countercharges of S1 to S3 segments to the voltage-gating of ion channels.

### Homology of Voltage Sensor Domains: Positively and Negatively Charged Residues

Voltage-gated channels show considerable homology in S1–S4 segments that comprise the voltage sensor domain (VSD). Alignments for a sample of voltage-gated sodium, calcium, and potassium channel VSDs are shown in **Figure 1**. Loci of conserved negatively charged (acidic) or polar residues are observed in S1–S3 segments. These residues represent putative countercharges to conserved, positively charged residues observed in the S4 segment.

**Figure 2:**
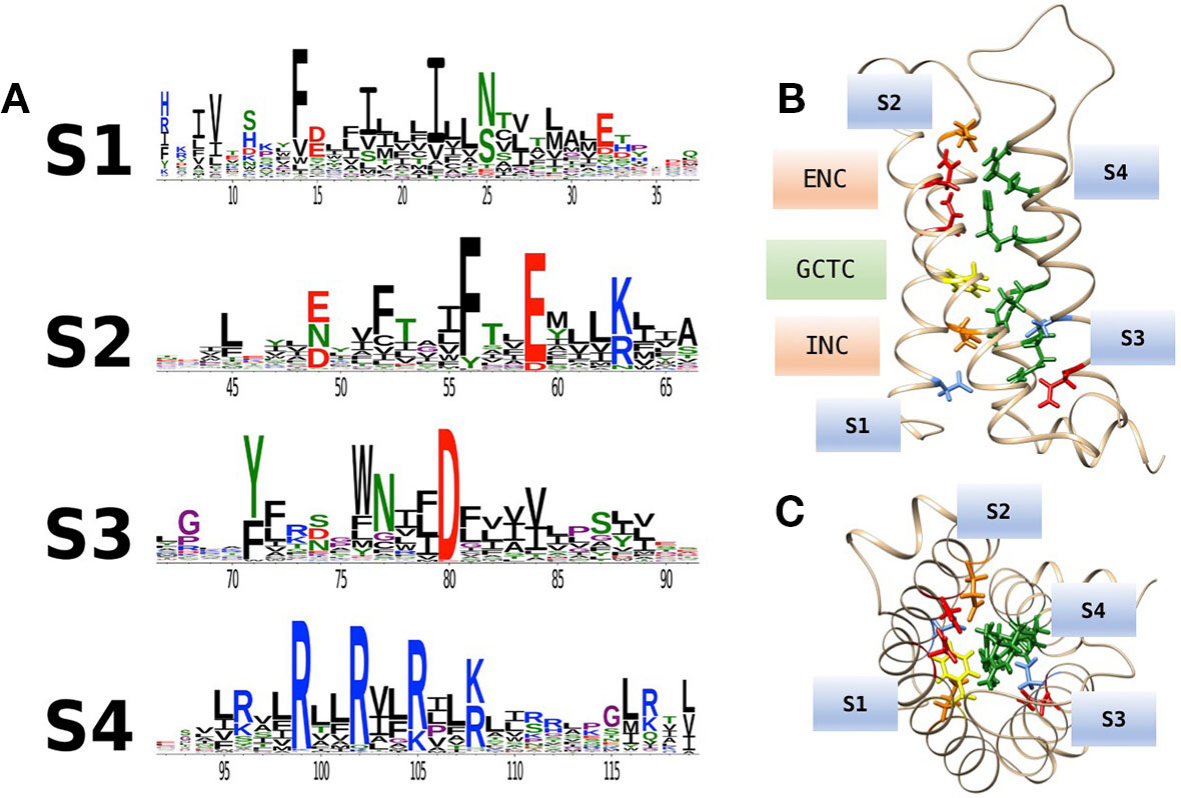
**(A)** Conservation of amino acid residues in voltage sensor domains (VSDs) across taxa, highlighting the evolutionary conservation of polar or acidic residues in S1–S3 (putative countercharges), positively charged arginine or lysine residues in S4, and conserved aromatic residues. With permission from J. Gen. Physiol. and first author (Palovcak et al., 2014). **(B)** Homology model of hNa_V_1.4 domain IV VSD based on prokaryotic structural information [3RVY.pdb, (Payandeh et al., 2011)] showing locations of consensus S1–S3 countercharges in the extracellular negatively charged (ENC) and intracellular negatively charged (INC) regions. A conserved aromatic in S2 (yellow) is also shown as part of the gating charge transfer center (GCTC). **(C)** Top view of the VSD showing side chains of S1–S3 countercharges facing the S4 arginine guanidyl groups.

An extensive analysis of VSDs from over 6,500 sequence alignments across taxa (**Figure 2A**; Palovcak et al., 2014) highlights the conservation of acidic or polar charges in S1–S3, positive charges in S4, and the degree of sequence identity. **Figure 2B** shows the location of conserved countercharges in relation to a central hydrophobic constriction site (HCS) that separates the outer and inner vestibules of the VSD. Countercharges above the HCS are located in an extracellular negatively charged region (ENC), with those nearer to the cytoplasmic region located in the intracellular negatively charged region (INC). Also shown is a conserved S2 aromatic residue located in a putative, gating charge transfer center (GCTC); this aromatic residue may act as a steric barrier to S4 translocation (Tao et al., 2010; Schwaiger et al., 2013). Current models of that S4 movement postulate that S4 arginine residues pass through an aqueous gating pore in the HCS region of the VSD [reviewed by (Groome et al., 2018)]. Side chains of S1–S3 countercharges face toward the guanidyl groups of the conserved S4 arginine residues (**Figure 2C**). The high degree of conservation of countercharge residues suggests that they play an important role or roles in channel function. Interactions of these countercharges with S4 residues have been investigated in a set of functional experiments described below, that support roles for countercharges in protein folding and S4 translocation.

### VSD S1–S3 Negative Charges Contribute to Voltage-Gating: Functional Studies

Experiments to describe gating charge movements associated with sodium channel function in the squid giant axon (Armstrong and Bezanilla, 1973; Armstrong and Bezanilla, 1977) were followed by characterization of gating charge movement in *Shaker* potassium channels (Perozo et al., 1992; Perozo et al., 1994; Bezanilla et al., 1994). The biophysical basis for displacement of the gating charge, and thus voltage-sensitivity, is S4 translocation in response to altered membrane potential [reviewed by (Bezanilla, 2000; Bezanilla, 2018)]. Investigations of the role of the S4 segment as the voltage sensor have employed mutagenesis including mutant cycle analysis, toxins to trap resting or activated states, thiosulfonate reagents to determine S4 residue accessibility, fluorescence measurements, and molecular dynamics simulations [reviewed by (Groome, 2014)]. Pertinent to this review, a series of investigations have supported the premise that sequential, salt-bridge (countercharge/S4 residue) interactions facilitate transitions from the resting to fully activated state of VGICs. Here, we describe the functional experiments that provide support for that role of countercharges in voltage-gating.

### Pairwise Electrostatic Interactions of Countercharges and S4 Residues: Potassium Channels

Countercharge residues that interact with S4 arginines were first identified in potassium channels [**Table 1**, and reviewed by (Fedida and Hesketh, 2001; Papazian et al., 2002; Kuang et al., 2015)]. In a series of papers, Papazian and colleagues showed that electrostatic interactions between S4 residues and negative countercharges in S2 and S3 were crucial in the folding or maturation of the fully functional tetrameric *Shaker* channel (Papazian et al., 1995; Seoh et al., 1996; Tiwari-Woodruff et al., 1997; Schulteis et al., 1998). Protein maturation in *Shaker* was dependent on short-range electrostatic interactions between S2 ENC residue E283 and S4 residues R368 (R3) and R371 (R4), and between INC residues E293 (S2) and D316 (S3) and S4 residue K374 (K5).

**Table 1:** Specific countercharge/S4 residue interactions identified in potassium channels.

Channel	Countercharge locus	S4 locus	Experimental approach	Functional interaction	Reference
*Shaker*	S2 ENC (E283)	R3 (R368) R4 (R371)	Charge swapping	Folding (maturation)	Tiwari-Woodruff et al., 1997
	S2 INC (E293) S3 INC (D316)	K5 (K374)	Charge swapping	Folding (maturation)	Papazian et al., 1995 Tiwari-Woodruff et al., 1997
	S2 ENC (E283)	R3 (R368) R4 (R371)	Charge swapping	Intermediate (deactivated) state Activated state	Tiwari-Woodruff et al., 2000
	S2 ENC (E283)	R1 (R362)	Double mutations, omega current	Resting state	Tombola et al., 2005 Tombola et al., 2007
EAG	S2 ENC (D274)	R3 (R353) R4 (R356)	Ni^2+^ inhibition	Activated state	Silverman et al., 2003
hERG	S1 INC (D411)	K5 (K538)	Mutant cycle analysis	Intermediate (deactivated) state	Zhang et al., 2005 Dou et al., 2017
	S2 ENC (D456)	K1 (K525)	Mutant cycle analysis	Intermediate (deactivated) state	Zhang et al., 2005

For each channel, residues are identified in ENC (extracellular negatively charged) or INC (intracellular negatively charged) regions of S1–S3 segments, locus in S4 segment, and with interpreted role of their paired interaction.

Comparison of the effects of single (charge-reversing) *versus* double (charge-swapping) mutations reveled pairwise interactions in the functional *Shaker* channel between E283 and R368 (intermediate, closed state activation) and between E283 and R371 [open-state activation; (Tiwari-Woodruff et al., 2000)]. A closed, resting state interaction was suggested between E283 and R362 (R1) by comparing the amplitude of omega current in single and double mutations using an R1S construct (Tombola et al., 2005; Tombola et al., 2007). Studies of EAG potassium channels confirmed interactions of countercharge and S4 arginine residues during activation. Silverman et al. (2003) showed that a unique, inhibitory effect of divalent ion (Ni^2+^) on the S2 ENC mutant D274A was reversed by pairing that mutant with R353Q (R3). Pairing D274A with R356Q (R4) did not reverse Ni^2+^ inhibition. Finally, the observation that Ni^2+^ inhibition was reversed in the triple D274A/R353Q/R356Q mutation suggested stepwise translocation of R353 and R356 to promote their interaction with D274. These results supported a two-stage model of S4 activation mediated by interaction of an S2 ENC residue with S4 arginine residues R3 and R4, as in *Shaker* channels.

In studies of human EAG channels (hERG), mutant cycle analysis showed that the S1 INC countercharge D411 and S2 ENC countercharge D456 interact with S4 residues K538 (K5) and K525 (K1), respectively, dictating early closed-state transitions (Zhang et al., 2005; Dou et al., 2017). Results using a similar approach suggested that S2 and S3 countercharges D460 and D509 may influence S4 translocation later in the activation pathway (Liu et al., 2003; Zhang et al., 2005). Taken together, results from these studies on potassium channels were consistent with a model of voltage-gating in which, from a resting conformation with R1 in the GCTC (Lin et al., 2011), sequential pairwise interactions of countercharge residues with S4 residues mediate channel activation.

### Pairwise Electrostatic Interactions of Countercharges and S4 Residues: Sodium Channels

Cloning of the prokaryotic sodium channel NaChBac (Ren et al., 2001) provided an advantage to study specific electrostatic interactions in the VSD of sodium channels. Like potassium channels, functional NaChBac is comprised of a homotetramer from the channel gene coding for one domain, providing robust effect with mutagenesis and more direct interpretation of the effect of these mutations on channel function.

In studies with NaChBac, cysteine substitution of countercharge and S4 residue pairs allowed for interpretation of their putative interaction (**Table 2**). For cysteine substitutions in close proximity, disulfide bond formation resulted in loss of channel availability. A key element of these experiments was the observation that reducing agents such as beta-mercaptoethanol or TCEP (tris 2-carboxyethyl phosphine) were able to rescue channel function by breaking the disulfide linkage. For mutations in which partial loss of channel opening was observed, interactions were determined from results showing that oxidizing agents such as hydrogen peroxide more fully abolished sodium currents, and with subsequent treatment with reducing agents that restored channel function. Double-mutant cycle analysis of the free energy change required for activation was used in these experiments to corroborate interactions [for review see (Bosshard et al., 2004)].

**Table 2:** Specific countercharge/S4 residue interactions identified in NaChBac sodium channels.

Channel	Countercharge locus	S4 locus	Experimental approach	Functional interaction	Reference
NaChBac	S2 ENC (D60)	R3 (R119)	Cysteine substitution disulfide locking	Activated state	DeCaen et al., 2008
	S2 ENC (D60) S2 INC (D70)	R4 (R122)	Cysteine substitution disulfide locking	Intermediate Activated states	DeCaen et al., 2009
	S1 ENC (E43)	T0 (T110) R1 (R113)	Cysteine substitution disulfide locking	Resting state	DeCaen et al., 2011
	S1 ENC (E43)	R2 (R116) R3 (R119)	Cysteine substitution disulfide locking	Intermediate Activated states	DeCaen et al., 2011
	S2 ENC (D60) S1 ENC (E43)	T0 (T110) R1 (R113) R2 (R116) R3 (R119) R4 (R122)	Cysteine substitution Mutant cycle analysis	Resting state Intermediate Activated states	Yarov-Yarovoy et al., 2012
	S1 ENC (E43)	R1 (R113) R2 (R116)	Mutant cycle analysis	Resting state Activated state	Paldi and Gurevitz, 2010

Residues are identified in extracellular negatively charged (ENC) or intracellular negatively charged (INC) regions of S1–S3 segments, locus in S4 segment, and with interpreted role of their paired interaction.

These experiments showed that NaChBac S2 residues D60 (ENC) and D70 (INC) interact with R119 (R3) and/or R122 (R4) during activation (DeCaen et al., 2008; DeCaen et al., 2009). Subsequent studies employing disulfide locking (DeCaen et al., 2011), and/or mutant cycle analysis (Paldi and Gurevitz, 2010; Yarov-Yarovoy et al., 2012) revealed interactions between a highly conserved ENC S1 residue (E43) and S4 residues T110 (T0), R113 (R1) in the resting state, and with R116 (R2), R119 (R3), and R122 (R4) during intermediate to fully activated states of the channel. A combined set of results was compared in Rosetta models illustrating the pairwise interaction of countercharge/S4 residue interactions from resting to activated states (Yarov-Yarovoy et al., 2012), with an interpretation of VSD gating similar to that suggested by the aforementioned experiments in potassium channels.

### Scanning Mutagenesis of Countercharge Mutations in Eukaryotic Channels

**Figure 3:**
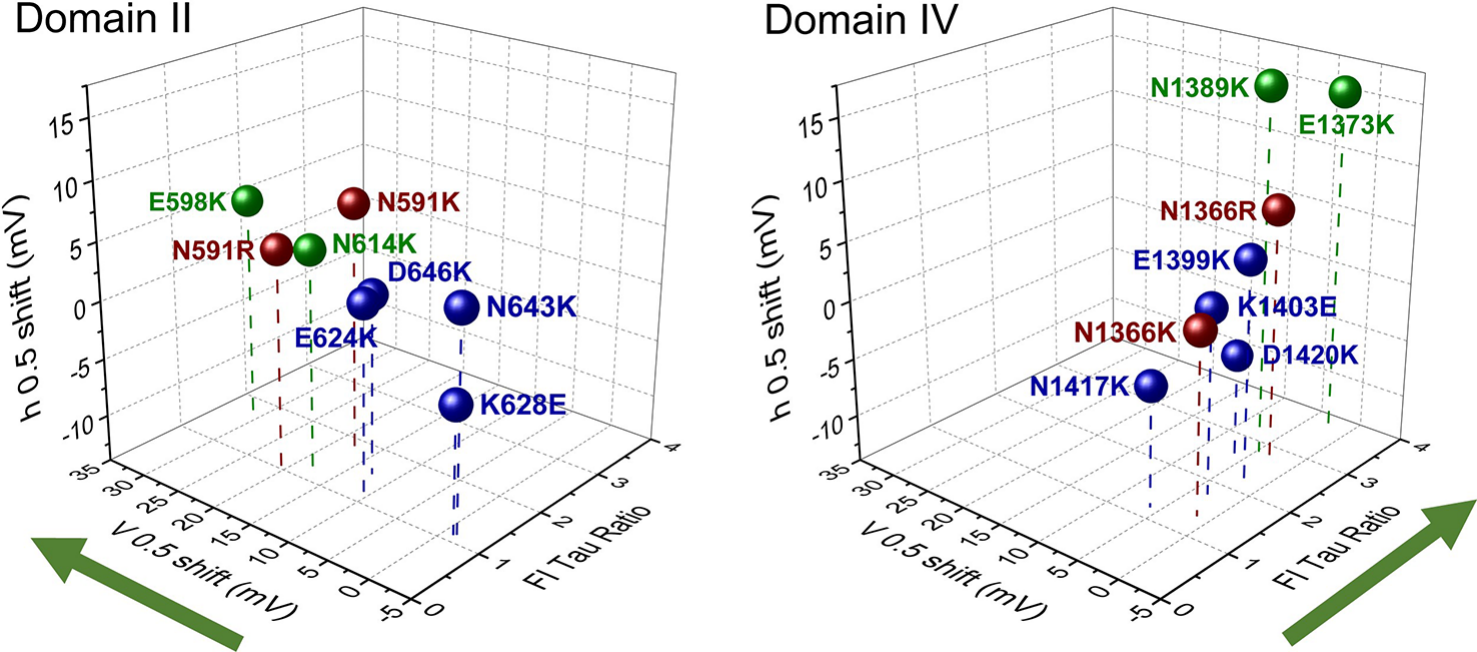
Scanning mutagenesis of putative countercharges in domains II and IV of the skeletal muscle sodium channel Na_V_1.4. Charge-reversing mutations in domain II decrease activation probability (indicated by shift in V_0.5_), while those in domain IV slow the entry of channels into a fast-inactivated state (indicated by the ratio of time constant (tau) of mutation with respect to that of wild type, at 20 mV). These effects are consistent with the hypothesis that countercharges facilitate certain domain-specific functions highlighted by green arrows (DII activation, DIV fast inactivation). Adapted from Groome and Winston (2013). Colors indicate countercharge loci as extracellular negatively charged (ENC) (green, red), or intracellular negatively charged (INC) (blue).

With the reports of crystal structures for prokaryotic sodium channels (Payandeh et al., 2011; Payandeh et al., 2012; Zhang et al., 2012) supporting the functional characterization of countercharge/S4 residue interactions in NaChBac, research attention was given to eukaryotic sodium channels. Here, both molecular dynamics simulations (Gosselin-Badaroudine et al., 2012) and scanning mutagenesis (Groome and Winston, 2013; Pless et al., 2014) probed each of the four domains of the skeletal muscle sodium channel (Na_V_1.4) for domain-specific roles of countercharges in activation and fast inactivation. Groome and Winston (2013) found that effects on activation probability were notable for mutations in domain II, while effects on fast inactivation kinetics were characteristic in domain IV (**Figure 3**). Using unnatural amino acid substitutions to precisely target the relative impact of charge content *versus* structure for gating contributions of countercharge residues, Pless et al. (2014) showed that mutations of ENC residues in domains I and II produce effects dependent on charge, whereas the effects of INC mutations are independent of charge alteration. These results were similar to those observed for *Shaker* channel mutations at ENC *versus* INC loci (Pless et al. (2011), and these authors presented the possibility that INC charges influence S4 movement through hydration of the inner vestibule of the VSD. Clearly, the significant differences observed in the functional effects of S1 to S3 mutations across domains, or at different loci (ENC *versus* INC) should be considered in the interpretation of the role(s) of countercharges in voltage-gating.

### Countercharges in Calcium and Proton Channels

Domain specific roles for voltage sensors in calcium channels have also been identified (Pantazis et al., 2014; Tuluc et al., 2016a). One source of functional variability across domains in Ca_V_ channels may be related to an outer countercharge observed in DIIS3 and DIVS3 segments (Coste de Bagneaux et al., 2018). In Ca_V_1.1, aspartate D1196, identified as D4 in the extracellular region of DIVS3, interacts with DIVS4 arginines R1 and R2, albeit differentially in distinct splice variants (Tuluc et al., 2016b). Specifically, the alternative splice variant Ca_V_1.1e excluding 19 amino acids in the DIVS3-S4 linker enhances activation, suggesting that in Ca_V_1.1a, the DIVS3-S4 linker disrupts D4 interaction with R1 and R2. Differential effects of DIVS3 D4 mutations in Ca_V_1.2 and Ca_V_1.3 are not solely dependent on the intact linker (Coste de Bagneaux et al., 2018) but also support a role for this countercharge in channel gating, and provide insight into domain-specific voltage-sensitivity in these channels.

VSD S1–S3 negative charges have roles in gating and selectivity in the voltage-gated proton channel H_V_1. Countercharge/S4 residue interactions during H_V_1 gating are suggested by mutant cycle analysis of the effects of single and double cysteine mutations (Chamberlain et al., 2014), and molecular dynamics simulations have defined salt-bridge interactions between countercharge and S4 residues during H_V_1 activation (Gianti et al., 2016). Interestingly, mutation of negative charge D112 in S1 results in channels that are non-functional (D112V), that display a shift in permeability to anions (i.e., D112H), or that maintain proton selectivity (D112E; Musset et al., 2012). The proton selectivity function of D112 might be based on its salt-bridge formation with S4 residue R211 (Chamberlain et al., 2015; Dudev et al., 2015). D112 and other countercharges in Hv1 S1–S3 appear to facilitate proton conduction through a mechanism in which proton binding sites are comprised of three pairs of acidic countercharges, with conformational change in the VSD promoting exchange of protons between sites (van Keulen et al., 2017). Proton-mediated disruption of the cation-pi interaction of S2 aromatic residue F150 with R211 has been proposed as a key determinant in the closed to open transition of the channel, facilitating the S2 E153 to S1 D112 proton exchange across the VSD permeation pathway.

### Structural Studies Illuminate Voltage Sensor Topology and Electrostatic Interactions

Following the successful crystallization of the KcSA potassium channel (Doyle et al., 1998), efforts to determine the atomic structure of VGICs intensified, and led to alternative hypotheses about the mechanism through which S4 voltage-sensing segments promote channel activation. The first full length structure of a voltage-gated potassium channel (K_V_AP) was solved with crystallography at a resolution of 3.2 Å (Jiang et al., 2003). In addition, an isolated VSD was solved at a resolution of 1.9 Å and whose structure revealed a salt-bridge between the S4 residue R133 (R5) with the S2 ENC countercharge D62. Importantly, the authors hypothesized that the second helical region in S3, along with the S4 helix, formed a voltage-sensor paddle that moved through the lipid membrane in response to membrane depolarization. Crystallization of the eukaryotic potassium channel K_V_1.2 (Long et al., 2005a; Long et al., 2005b) and the chimera K_V_1.2/2.1 (Long et al., 2007) partially reconciled the paddle hypothesis of charge displacement with functional data that supported vertical displacement of the S4 helix [reviewed by (Horn, 2002; Gandhi and Isacoff, 2002; Elinder et al., 2007)]. As described later, structural data for K_V_ VSDs were utilized in molecular dynamics simulations (Delemotte et al., 2011; Jensen et al., 2012) that supported a mechanism of voltage-gating dependent on multiple salt-bridge interactions facilitating intermediate states of channel activation.

The crystal structure of the prokaryotic sodium channel Na_V_Ab (Payandeh et al., 2011) revealed the proximity of ENC and INC countercharges with specific S4 residues. Importantly, Kv1.2/2.1 and Na_V_Ab structures showed the S4 segment in nearly identical positions (Long et al., 2007; Payandeh et al., 2011), confirming the vertical position of the S4 segment in the activated state. Countercharge to S4 arginine residue interactions have been revealed with crystallographic data from Na_V_Ab, Na_V_Rh, and Na_V_Ms (Payandeh et al., 2011; Payandeh et al., 2012; Zhang et al., 2012; Sula et al., 2017; Wisedchaisri et al., 2019). It has also been hypothesized that negatively charged residues tune the hydrophilicity of the inner and outer vestibules of the VSD (Palovcak et al., 2014), while the central, hydrophobic region separates these vestibules and focuses the electric field (Starace and Bezanilla, 2004; Ahern and Horn, 2005; Chanda and Bezanilla, 2008; Lacroix et al., 2014).

Recently, improvements in cryo-electron microscopy (cryo-EM) have allowed for rapid advances in structure determination of large membrane proteins, including eukaryotic sodium and calcium channels. Shen et al. (2017) used cryo-EM to determine the structure of Na_V_PaS, the cockroach isoform of Na_V_1.4, at 3.8 Å. Shortly thereafter, the structure of EeNa_V_1.4, the electric eel construct of Na_V_1.4, was solved to 4.0 Å and in complex with the accessory β1 subunit (Yan et al., 2017). Many of the countercharge/S4 residue interactions described in the structures of prokaryotic sodium channels are reiterated in these eukaryotic structures, and in the cryo-EM structure of Ca_V_1.1 (Wu et al., 2016). When the structure of the human Na_V_1.4 channel/β1 subunit was solved to 3.2 Å (Pan et al., 2018), an expanded set of interactions between S1 and S3 countercharges and S4 residues was presented, and suggested a role for polar residues in stabilizing VSDs across domains. Together, these cryo-EM structures have provided a structural framework to complement functional investigations of domain-specific functions of eukaryotic VSDs (Chanda and Bezanilla, 2002; Goldschen-Ohm et al., 2013; Tuluc et al., 2016a) and of domain-specific effects of S1–S3 countercharge mutations (Groome and Winston, 2013; Pless et al., 2014).

### Molecular Dynamics Simulations Bridge Structural and Functional Work

Molecular dynamics simulations have been used extensively in computational approaches to investigate ion channel function, by providing a view of time-dependent interactions within the VSD in response to applied membrane potential on the system [for reviews see (Delemotte et al., 2009; Cournia et al., 2015; Howard et al., 2018)]. These mathematical simulations solve the equations for motion in three dimensions for atoms with a defined mass and charge. As parameters such as membrane potential and amino acid sequence can be easily manipulated, these simulations allow researchers to test specific hypotheses about countercharge interactions with S4 residues, as the S4 segment vertically transverses the electric field. However, these simulations are computationally limited to short timescales, making it difficult to simulate an entire conformational transition.

Molecular dynamics simulations of VSDs conducted following the publication of voltage-gated potassium channel structures (Jiang et al., 2003; Long et al., 2005a; Long et al., 2005b; Long et al., 2007) probed the activated state of the voltage sensor. Several research groups were interested in hydration of the intracellular and extracellular vestibules, as well an interaction between the voltage sensor and surrounding lipid environment. Treptow and Tarek (2006) found that the outer S4 arginine residues are accessible to water in the activated state, and Jogini and Roux (2007) proposed that S4 arginine residues interact with both negatively charged lipid phosphate groups and S2 countercharges. While their continuum electrostatic calculations showed that the membrane potential varies greatly across the membrane, the greatest membrane potential was focused on the center of the voltage sensor, a hypothesis that has been supported functionally (Starace and Bezanilla, 2004; Ahern and Horn, 2005). Simulations of S4 dynamics in response to an applied membrane potential suggested that the S4 segment transverses the membrane in a screw-like motion (Nishizawa and Nishizawa, 2008), with salt-bridges (Treptow et al., 2009) and hydrogen bonding networks (Bjelkmar et al., 2009) stabilizing intermediate states. These simulations supported a model of vertical translocation of the S4 helix through the membrane, and groups began focusing on defining the intermediate states of the voltage sensor and interactions that occurred within each state.

Due to computational limitations, simulating each intermediate state of the VSD required biasing simulations based on initial starting structure (Yarov-Yarovoy et al., 2006; Wood et al., 2012), a directed path of S4 helix motion through the membrane (Delemotte et al., 2011; Schwaiger et al., 2011) or enhanced sampling techniques (Delemotte et al., 2015). Resting state models have been built by multiple groups. Yarov-Yarovoy et al. (2006); Pathak et al. (2007), and Henrion et al. (2012) used a *de novo* and homology modeling approach, Delemotte et al. (2011) used a combination of harmonic restraints and steered molecular dynamics, Vargas et al. (2011) computed an average structure over multiple simulations, and Jensen et al. (2012) ran a long time-scale simulation to reach a resting state structure. Although the methodologies to produce them were unique, these models showed a high degree of similarity, with the S4 helix rotated and translated inwardly, and R1 located between the S2 ENC and INC countercharges, and near the conserved S2 aromatic residue of the GCTC. A consensus model of the resting state was then proposed (Vargas et al., 2012). While simulations have suggested that the S4 segment adopts a 3_10_ helical secondary structure to favor the resting state and align S4 arginine side chains with those of negative countercharges (Villalba-Galea et al., 2008; Khalili-Araghi et al., 2010; Schow et al., 2010; Schwaiger et al., 2011), the functional contribution of 3_10_ helical structure to VGIC gating remains to be clearly elucidated (Kubota et al., 2014; Bassetto et al., 2019).

Computational approaches to VSD activation have confirmed and refined the description of intermediate states. Nishizawa and Nishizawa (2008) initially hypothesized that the free energy landscape between intermediate states would be “ragged,” indicating multiple pathways of transition between states due to the large number of electrostatic interactions taking place. This hypothesis was confirmed by Delemotte et al. (2015), who characterized the transition between the resting and a partially activated state using well-tempered metadynamics. They showed that R1 must transit across the hydrophobic septum prior to the transition of R3 engaged with intracellular lipid phosphate groups, to R3 engaged with the INC S2 countercharge. Although this single most favorable pathway between a resting and intermediate state was found, their results did show a rough free energy landscape, as previously hypothesized. However, when the free energy was plotted as a function of gating charge, a smooth free energy landscape was produced, with two well-defined energy wells corresponding to the resting and intermediate states. It is important to note that the bulk of these simulations were completed using the Kv1.2 channel as a model. Thus, although many of these findings can be translated to other channel types, it is yet unknown how the differences between VSDs affect dynamics. For example, the cryo-EM structure of hNa_V_1.4 (Pan et al., 2018) suggests unique electrostatic interactions within each VSD, and differences in the extent of voltage sensor activation across domains.

**Figure 4:**
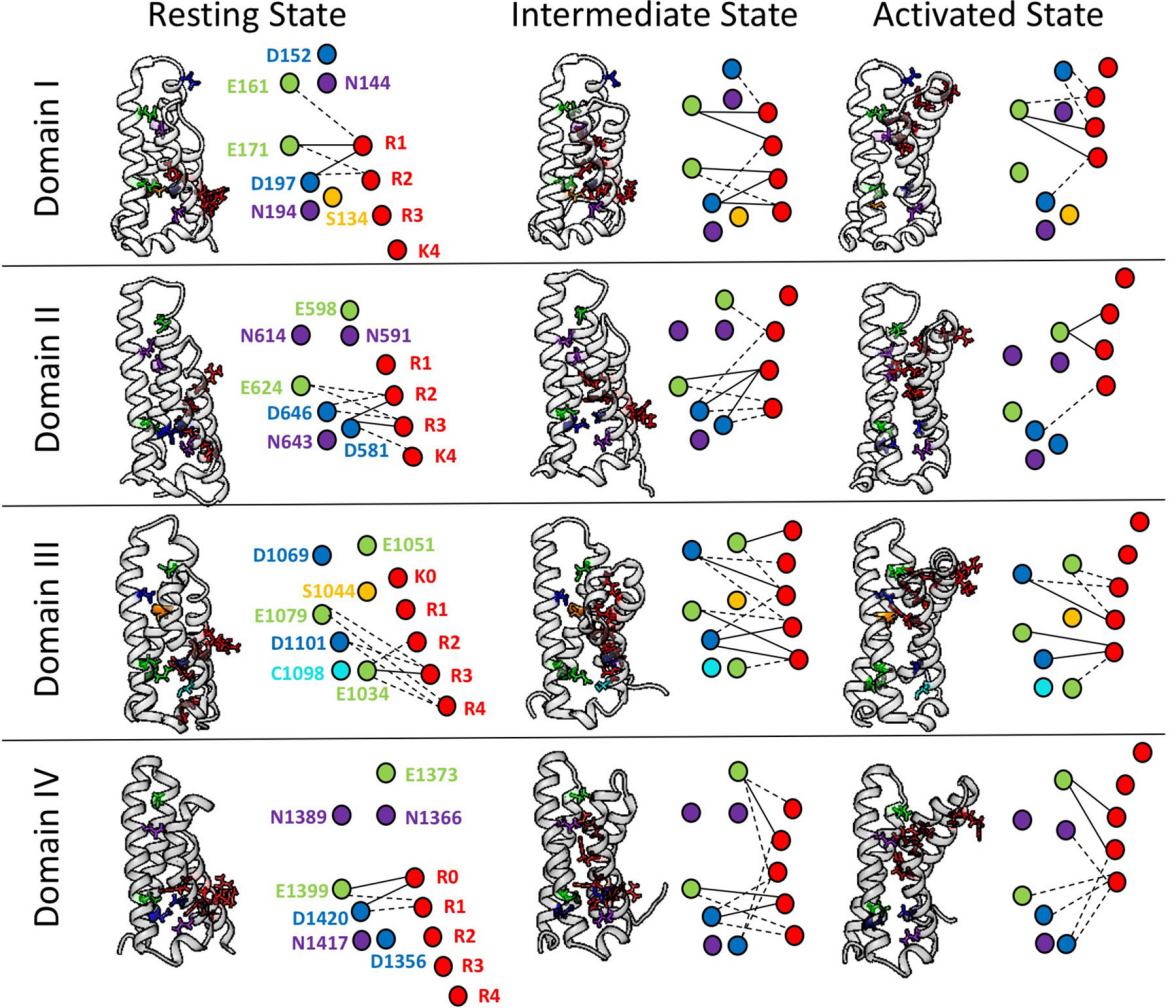
Electrostatic interactions between countercharges (aspartate, blue; glutamate, green; asparagine, purple; serine, yellow, cysteine, cyan) and each of four to five positively charged residues in S4 segments (arginine or lysine, red) in domains I–IV of the skeletal muscle sodium channel Na_V_1.4. Nodes indicate charged and polar residues within the voltage sensor, while edge width indicates strength of interaction. The profile of interactions is given for a presumptive resting state, intermediate closed state, and activated state. Reproduced with permission from Idaho State University Libraries (Bayless-Edwards, 2019).

To further explore the differences in interactions between domains of hNa_V_1.4, we have conducted a network analysis from short time-scale molecular dynamics simulations (Bayless-Edwards, 2019). **Figure 4** shows each S4 segment translocated intracellularly from an initial activated structure (Pan et al., 2018) to produce both an intermediate state and a potential resting state structure, using methods similar to those of Wood et al. (2012). gRINN (get Residue Interaction eNergies and Networks) was used to complete the network analysis (Sercinoglu and Ozbek, 2018). Our results support the presence of unique electrostatic interactions in each domain over multiple conformations. The strongest interactions are between acidic countercharges with S4 residues, consistent with other simulations (Gosselin-Badaroudine et al., 2012) and functional experiments in sodium channels (Groome and Winston, 2013 **; Pless et al., 2014).

### Water Permeability Within and Across the Voltage Sensor Domain

Solvation of the VSD is an essential component in models of voltage-gating in which positively charged S4 arginine residues traverse the membrane through an aqueous environment (Freites and Tobias, 2015). Water permeation within the VSD is often measured by calculating either the solvent-accessible surface or the simulated average water occupancy along the z-axis of the membrane. As is expected by the hourglass shape of the VSD comprising inner and outer vestibules, water occupancy is highest at the edges of the membrane and decreases to the hydrophobic septum (Freites et al., 2006; Schow et al., 2010; Gosselin-Badaroudine et al., 2012). Chakrapani et al. (2010) used site-directed spin-labeling to quantify the hydrophilicity of outer and inner vestibules of the VSD and incorporated these environmental restraints into molecular dynamics simulations of NaChBac. Their results showed that S4 arginines R1 and R2 move through a hydrophilic environment toward their interaction with phospholipid heads of the bilayer as the S2 ENC (D60)/S4 R3 salt-bridge interaction is formed with activation of the channel. The significance of hydration in voltage-gating is further emphasized with the finding that S4 aspartate substitutions in the *Shaker* channel are remarkably tolerated with respect to gating function (Diaz-Franulic et al., 2018), and by the corollary observation that mutation of S1–S3 residues comprising the hydrophobic plug have dramatic effects on gating charge movement (Lacroix et al., 2014, and reviewed by Bezanilla, 2018).

In diseases such as hypokalemic periodic paralysis and dilated cardiomyopathy, mutation of S4 arginines can lead to permeation of protons or cations between the inner and outer vestibules of the VSD, a finding supported using both molecular dynamics simulations (Jensen et al., 2012; Monteleone et al., 2017) and cryo-EM (Jiang et al., 2018). This omega current is a characteristic effect of mutations in these diseases, and is a significant factor in pathogenesis [reviewed by Groome et al., 2018)]. Interestingly, the width of the hydrophobic region varies between sodium channel domains (Gosselin-Badaroudine et al., 2012), and the large hydrophobic septum in DIV may explain the lack of observed omega current associated with DIVS4 channelopathy mutations (Francis et al., 2011), even with periodic paralysis as a contributing element to the phenotype (Poulin et al., 2018).

It has been hypothesized that mutations near the hydrophobic septum disrupt not only steric inhibition of water molecules and ions (Jiang et al., 2018), but also hydrogen bonding (Monteleone et al., 2017; Poulin et al., 2018) and electrostatic interactions (Bayless-Edwards et al., 2018). This hypothesis is consistent with the premise that charged and polar residues play an important role in the hydration of ion channel vestibules in VSDs (Pless et al., 2011; Palovcak et al., 2014). An interesting set of questions remain as to the nature and extent of contributions of polar, countercharge residues to specific aspects of voltage-gating. For example, mutations of VSD polar residues do elicit substantial effects on channel activation, fast inactivation, and slow inactivation (Gamal El-Din et al., 2013; Groome and Winston, 2013; Pless et al., 2014; Groome et al., 2019). The domain-specific VSD conformations underlying these channel functions are an important target for investigation (Yarov-Yarovoy and DeCaen, 2019) and a determination of the impact of hydration provided by polar countercharges in voltage-gating may provide some unique insight.

### Countercharge Mutations in Disease

Inherited mutations in the genes for VGICs comprise a set of channelopathies including epilepsy and migraine syndromes, pain, cardiac arrhythmia syndromes, myotonia, periodic paralysis, and congenital myasthenia (for reviews see Catterall, 2012; Bezzina et al., 2015; Cannon, 2015). These mutations may result in a truncated protein, disrupt trafficking or assembly with accessory subunits, or produce gating defects. Channelopathy mutations have been studied in heterologous expression to characterize their biophysical impact on membrane excitability, and in transgenics to test their impact on physiology and behavior [reviewed by (Cannon, 2018; Catterall, 2018)].

A number of variants predicting VSD countercharge mutations associated with disease phenotypes have been identified, with missense mutations for these channelopathies given in **Table 3**. Noteworthy on this list are the prevalence of SCN1A mutations identified in genetic screening of patients with intractable epilepsy syndromes. These countercharge mutations have yet to be functionally characterized in heterologous expression (Brunklaus et al., 2020). However, several countercharge mutations identified in cardiac arrhythmia or skeletal muscle syndromes have been characterized, and are described here.

**Table 3:** Voltage-gated ion channel (VGIC) countercharge S1–S3 mutations identified in human disease phenotypes.

Gene	S1 mutation	S2 mutation	S3 mutation	Phenotype	Reference
SCN1A			DI: N191K/Y	EIEE6	Depienne et al., 2009; Depienne et al., 2010 Huang et al., 2017
			DI: D194G/N	EIEE6	Depienne et al., 2009 Azmanov et al., 2010 Kodera et al., 2013
	DIII: E1221K			EIEE6	Zuberi et al., 2011
	DIII: S1231R/T			EIEE6	Fujiwara et al., 2003 Kearney et al., 2006
	DIII: E1238D			EIEE6	Harkin et al., 2007
		DIII: E1266A		EIEE6	Zuberi et al., 2011
			DIII: D1288N	EIEE6	Zuberi et al., 2011
	DIV: D1544A/G			EIEE6	Depienne et al., 2009 Zuberi et al., 2011
	DIV: E1561K			EIEE6	Depienne et al., 2009
			DIV: N1605I/S	EIEE6	Parrini et al., 2017 Zuberi et al., 2011
			DIV: D1608G/Y	EIEE6	Wang et al., 2012 Marini et al., 2007
SCN2A	DI: N132K			EIEE11	Matalon et al., 2014
		DI: E169G		EIEE11	Nakamura et al., 2013 Parrini et al., 2017
	DIII: E1211K			EIEE11	Ogiwara et al., 2009 Wong et al., 2015
SCN4A		DIII: D1069N		CM	Zaharieva et al., 2016
	DIV: N1366S			PMC	Ke et al., 2017
SCN5A		DI: E161K/Q		BRGDA/PFHB	Smits et al., 2002; Smits et al., 2005 Meregalli et al., 2009 Kapplinger et al., 2010
	DIII: E1225K			BRGDA/LQT-3	Smits et al., 2002 Tester et al., 2005
		DIII: D1243N		BRGDA	Kapplinger et al., 2010
		DIII: E1253G		BRGDA	Kapplinger et al., 2010
			DIII: D1275N	BRGDA/AS/DCM	Groenewegen et al., 2003 Hayano et al., 2017
	DIV: N1541D			BRGDA/AF/SND	Dharmawan et al., 2019
	DIV: E1548K			BRGDA	Kapplinger et al., 2010
		DIV: E1574K		BRGDA	Kapplinger et al., 2010
			DIV: D1595N/H	DCM/SND/AV Block	Wang et al., 2002 McNair et al., 2004 Nguyen et al., 2008
KCNQ1	C136F			LQT-1	Tester et al., 2005
		E160K/V		LQT-1	Splawski et al., 2000 Tester et al., 2005 Kapplinger et al., 2009
			S199A	LQT-1	Kapplinger et al., 2009
			D202H/N	LQT-1	Napolitano et al., 2005 Kapplinger et al., 2009
KCNH2	T421M			LQT-2	Tester et al., 2005 Kapplinger et al., 2009
	S428L			LQT-2	Napolitano et al., 2005
		D456Y		LQT-2	Tester et al., 2005
		D466Y		LQT-2	Kapplinger et al., 2009
			D501H/N	LQT-2	Napolitano et al., 2005 Jongbloed et al., 2002 Kapplinger et al., 2009
KCNA1			N225D	HM	Glaudemans et al., 2009

EIEE, early infantile epileptic encephalopathy; CM, congenital myopathy; PMC, paramyotonia congenita; BRGDA, Brugada syndrome; PFHB, progressive familial heart block; LQT, long QT syndrome; AS, atrial standstill; DCM, dilated cardiomyopathy; SND, sinus node dysfunction; HM, hypomagnesemia.

### Functional Characterization of Countercharge Channelopathy Mutations

Channelopathy mutations identified in patients with Brugada syndrome and/or overlapping phenotypes such as dilated cardiomyopathy confer loss of function in the cardiac sodium channel hNa_V_1.5 (SCN5A; for reviews see Garcia-Elias and Benito, 2018; Asatryan, 2019). A few of these mutations are at countercharge loci, and for which biophysical characterization has been reported. Loss of function may be explained to some extent with observed reduction in current density, as in DIS2 ENC mutation E161Q (Meregalli et al., 2009). In contrast, N1541D (DIVS1 ENC) elicits specific gating defects including enhanced probability of fast inactivation, and slowed recovery from open-state fast inactivation (Dharmawan et al., 2019). Interestingly, each of these gating defects are reiterated in the homologous Na_V_1.4 mutation N1366D (Groome and Winston, 2013).

The SCN5A DIIIS3 INC mutation D1275N produces mild, loss of function gating defects including impaired activation and enhanced fast inactivation when studied in heterologous expression (Groenewegen et al., 2003; Hayano et al., 2017). However, D1275N promotes significant cardiac defects such as slowed conduction, heart block, atrial fibrillation, ventricular tachycardia, and dilated cardiomyopathy when inserted in transgenic mice (Watanabe et al., 2011) or zebrafish (Huttner et al., 2013). Two SCN5A channelopathy mutations have been characterized at D1595 (DIVS3 INC). First, D1595N has been linked to atrioventricular block (Wang et al., 2002). The mutation reduces current density and enhances fast and slow inactivation consistent with the cardiac muscle phenotype as shown in action potential modeling. Histidine substitution at this locus (D1595H) is identified in patients with arrhythmic dilated cardiomyopathy (Olson et al., 2005), and enhances fast inactivation (Nguyen et al., 2008).

In the skeletal muscle sodium channel hNa_V_1.4 (SCN4A), the mutation D1069N (DIIIS2 ENC) has been identified in family members presenting with congenital myopathy (Zaharieva et al., 2016). The mutation produces a right shift in the midpoint of the activation curve, consistent with loss of function (hypoexcitability). In contrast, N1366S (DIVS1 ENC), identified in a family diagnosed with paramyotonia congenita, produces gain of function effects including enhanced activation, slowed entry into the fast-inactivated state, and accelerated recovery (Ke et al., 2017). These effects are consistent with a hyperexcitable skeletal muscle fiber phenotype that characterizes the disease. Thus, functional characterizations of countercharge mutations have extended the genotype to phenotype correlation for loss or gain of function in SCN5A and SCN4A channelopathies.

### Countercharge Roles in Voltage-Gating: Concluding Remarks

Common sequence motifs in VSDs include the positively charged S4 region, and conserved aromatic, acidic, or polar amino acid residues in S1–S3 segments. Investigations of the mechanism of voltage-gating in VGICs have supported the hypothesis that VSD negatively charged residues act as countercharges to facilitate S4 translocation in an energetically unfavorable membrane environment. Functional experiments have revealed pairwise interactions between countercharge/S4 residues that play an important role in protein folding, the resting state of the channel, and in outward movement of the S4 segment in the steps leading to channel opening. Determinations of protein structure at atomic resolution supports such countercharge/S4 residue interactions, and have facilitated in-depth analyses of their choreography, using molecular dynamics simulations. These simulations have also been instrumental in present efforts to elucidate the mechanisms by which countercharge residues regulate VSD hydration and to explore the role of hydration in ion channel function. An expanding genetic database has revealed that countercharge mutations comprise a portion of published variants of ion channel genes in patients presenting with neural, cardiac, or skeletal muscle disorders. Genotype to phenotype correlation for countercharge mutations is an important aspect of continued research of the biophysical mechanisms of channelopathies.

## Author Contributions

JG and LB-E contributed equally to the writing of this review article.

## Funding

This work was supported by the NIH under 1R15NS093579-01A1 to JG and Idaho INBRE (through an Institutional Development Award from the National Institute of General Medical Sciences) under Grant P20GM103408.

## Conflict of Interest

The authors declare that the research was conducted in the absence of any commercial or financial relationships that could be construed as a potential conflict of interest.

## References

[B1] Ahern C. A. Horn R. (2005). Focused electric field across the voltage sensor of potassium channels. Neuron 48 (1), 25–29. 10.1016/j.neuron.22056.08.020 16202706

[B2] Armstrong C. M. Bezanilla F. (1973). Currents related to movement of the gating particles of the sodium channels. Nature 242 (5398), 459–461. 10.1038/242459a0 4700900

[B3] Armstrong C. M. Bezanilla F. (1977). Inactivation of the sodium channel. II. Gating current experiments. J. Gen. Physiol. 70 (5), 567–590. 10.1085/jgp.70.5.567 591912 PMC2228472

[B4] Asatryan B. (2019). Cardiac sodium channel dysfunction and dilated cardiomyopathy: a contemporary reappraisal of pathophysiological concepts. J. Clin. Med. 8 (7), E1029. 10.3390/jcm8071029 31336969 PMC6678327

[B5] Azmanov D. N. Zhelyaskova S. Dimova P. S. Radionova M. Bojinova V. Florez L. (2010). Mosaicism of a missense SCN1A mutation and Dravet syndrome in a Roma/Gypsy family. Epileptic Disord. 12 (2), 117–124. 10.1684/epd.2010.0311 20562086

[B6] Bassetto C. A. Z. Jr. Carvalho-de-Souza J. L. Bezanilla F. (2019). Metal bridge in S4 segment supports helix transition in Shaker channel. Biophys. J. 117, 1–12. 10.1016/j.bpj.2019.08.035. 31635788 PMC7037500

[B7] Bayless-Edwards L. Winston V. Lehmann-Horn F. Arinze P. Groome J. R. Jurkat-Rott K. (2018). Na_V_1.4 DI-S4 periodic paralysis mutation R222W enhances inactivation and promotes leak current to attenuate action potentials and depolarize muscle fibers. Sci. Rep. 8, 10372. 10.1038/s41598-018-28594-5 29991727 PMC6039468

[B8] Bayless-Edwards L. (2019). From molecule to cell: using electrophysiology and multiscale modelling to understand how Na_V_1.4 controls electrical excitability. [Master’s thesis]. (Pocatello, ID: Idaho State University).

[B9] Bezanilla F. Perozo E. Stefani E. (1994). Gating of Shaker K+ channels: II. The components of gating currents and a model of channel inactivation. Biophys. J. 66 (4), 1011–1021. 10.1016/S0006-3495(94)80882-3 8038375 PMC1275808

[B10] Bezanilla F. (2000). The voltage sensor in voltage-dependent ion channels. Physiol. Rev. 80 (2), 555–592. 10.1151/physrev.2000.80.2.555 10747201

[B11] Bezanilla F. (2018). Gating currents. J. Gen. Physiol. 150 (7), 911–932. 10.1085/jgp.201812157 29941430 PMC6028497

[B12] Bezzina C. R. Lahrouchi N. Priori S. G. (2015). Genetics of sudden cardiac death. Circ. Res. 116 (12), 1919–1936. 10.1161/CIRCRESAHA.116.304030 26044248

[B13] Bjelkmar P. Niemela P. S. Vattulainen I. Lindahl E. (2009). Conformational changes and slow dynamics through microsecond polarized atomistic molecular simulation of an integral Kv1.2 ion channel. PLoS Computat. Biol. 5 (2), e1000289. 10.1371/journal.pcbi.1000289 PMC2632863 19229308

[B14] Bosshard H. R. Marti D. N. Jelesarov I. (2004). Protein stabilization of salt bridges: concepts, experimental approaches and clarification of some misunderstandings. J. Mol. Recognit. 17 (1), 1–16. 10.1002/jmr.657 14872533

[B15] Brunklaus A. Schorge S. Smith A. D. Ghanty I. Stewart K. Gardiner S. (2020). SCN1A variants from bench to bedside – improved prediction from functional characterization. Hum. Mutat. 41, 363–374. 10.1002/humu.23943. in press. 31782251

[B16] Cannon S. C. (2015). Channelopathies of skeletal muscle excitability. Compr. Physiol. 5 (2), 761–790. 10.1002/cphy.c140062 25880512 PMC4754081

[B17] Cannon S. C. (2018). “Sodium channelopathies of skeletal muscle,” in Voltage-gated sodium channels: structure, function and channelopathies, vol. 246 . Ed. Chahine M. Handbook of Experimental Pharmacology (Switzerland: Springer International Publishing AG), 309–330. 10.10017/164_2017_52 PMC5866235 28939973

[B18] Catterall W. A. (2012). Voltage-gated sodium channels at 60: structure, function and pathophysiology. J. Physiol. 590 (11), 2577–2589. 10.1113/jphysiol.2011.224204 22473783 PMC3424717

[B19] Catterall W. A. (2018). Dravet syndrome: a sodium channel interneuronopathy. Curr. Opin. Physiol. 2, 42–50. 10.1016/j.cophys.2017.12.007 30123852 PMC6091224

[B20] Chakrapani S. Sompornpisut P. Intharathep P. Roux B. Perozo E. (2010). The activated state of a sodium channel voltage sensor in a membrane environment. Proc. Natl. Acad. Sci. U. S. A. 107 (12), 5435–5440. 10.1073/pnas.0914109107 20207950 PMC2851821

[B21] Chamberlain A. Qiu F. Rebolledo S. Wang Y. Noskov S. Y. Larsson H. P. (2014). Hydrophobic plug functions as a gate in voltage-gated proton channels. Proc. Natl. Acad. Sci. U. S. A. 111 (2), E273–E282. 10.1073/pnas.1318018111 24379371 PMC3896152

[B22] Chamberlain A. Qiu F. Wang Y. Noskov S. Y. Larsson H. P. (2015). Mapping the gating and permeation pathways in the voltage-gated proton channel Hv1. J. Mol. Biol. 427 (1), 131–145. 10.1016/j.jmb.2014.11.018 25481746 PMC4381436

[B23] Chanda B. Bezanilla F. (2002). Tracking voltage-dependent conformational changes in skeletal muscle sodium channel during activation. J. Gen. Physiol. 120 (5), 629–645. 10.1085/jgp.20028679 12407076 PMC2229551

[B24] Chanda B. Bezanilla F. (2008). A common pathway for charge transport through voltage-sensing domains. Neuron 57 (3), 345–351. 10.1016/j.neuron.2008.01.015 18255028

[B25] Coste de Bagneaux P. Campiglio M. Benedetti B. Tuluc P. Flucher B. E. (2018). Role of putative voltage-sensor countercharge D4 in regulating gating properties of Cav1.2 and Cav1.3 calcium channels. Channels 12 (1), 249–261. 10.1080/19336950.2018.1482183 30001160 PMC6161609

[B26] Cournia Z. Allen T. W. Andricioaei I. Antonny B. Baum D. Brannigan G. (2015). Membrane protein structure, function and dynamics: a perspective from experiments and theory. J. Mem. Biol. 248 (4), 611–640. 10.1007/s00232-015-9802-0 PMC4515176 26063070

[B27] DeCaen P. G. Yarov-Yarovoy V. Zhao Y. Scheuer T. Catterall W. A. (2008). Disulfide-locking of a sodium channel voltage sensor reveals ion pair formation during activation. Proc. Natl. Acad. Sci. U. S. A. 105 (39), 15142–15147. 10.1073/pnas.0806486105 18809926 PMC2567506

[B28] DeCaen P. G. Yarov-Yarovoy V. Sharp E. M. Scheuer T. Catterall W. A. (2009). Sequential formation of ion pairs during activation of a sodium channel voltage sensor. Proc. Natl. Acad. Sci. U. S. A. 106 (52), 22498–22503. 10.1073/pnas.0912307106 20007787 PMC2799717

[B29] DeCaen P. G. Yarov-Yarovoy V. Scheuer T. Catterall W. A. (2011). Gating charge interactions with the S1 segment during activation of a Na+ channel voltage sensor. Proc. Natl. Acad. Sci. U. S. A. 108 (46), 18825–18830. 10.1073/pnas.1116449108 22042870 PMC3219111

[B30] Delemotte L. Dehez F. Treptow W. Tarek M. (2009). Modeling membranes under a transmembrane potential. J. Phys. Chem. B. 112 (18), 5547–5550. 10.1021/jp710846y 18412411

[B31] Delemotte L. Tarek M. Klein M. L. Amaral C. Treptow W. (2011). Intermediate states of the Kv1.2 voltage sensor from atomistic molecular dynamics simulations. Proc. Natl. Acad. Sci. U. S. A. 108 (15), 6019–6114. 10.1073/pnas.1102724108 PMC3076833 21444776

[B32] Delemotte L. Kasimova M. A. Klein M. L. Tarek M. Carnevale V. (2015). Free-energy landscape of ion channel voltage-sensor domain activation. Proc. Natl. Acad. Sci. U. S. A. 112 (1), 124–129. 10.1073/pnas.1416959112 25535341 PMC4291615

[B33] Depienne C. Trouillard O. Saint-Martin C. Gourfinkel-An I. Bouteiller D. Carpentier W. (2009). Spectrum of SCN1A gene mutations associated with Dravet syndrome: analysis of 333 patients. J. Med. Genet. 46 (3), 183–191. 10.1136/jmg.2008.062323 18930999

[B34] Depienne C. Trouillard O. Gourfinkel-An I. Saint-Martin C. Bouteiller D. Barthez-Carpentier M. A. (2010). Mechanisms for variable expressivity of inherited SCN1A mutations causing Dravet syndrome. J. Med. Genet. 47 (6), 404–410. 10.1136/jmg.2009.074328 20522430

[B35] Dharmawan T. Nakajima T. Iizuka T. Tamura S. Matsui H. Kaneko Y. (2019). Enhanced closed-state inactivation of mutant cardiac sodium channels (SCN5A N1541D and R1632C) through different mechanisms. J. Mol. Cell. Cardiol. 130, 88–95. 10.1016/j.yjmcc.2019.02.023 30935997

[B36] Diaz-Franulic I. Gonzalez-Perez V. Moldenhauer H. Navarro-Quezada N. Naranjo D. (2018). Gating-induced large aqueous volumetric remodeling and aspartate tolerance in the voltage sensor domain of Shaker K+ channels. Proc. Natl. Acad. Sci. U. S. A. 115 (32), 8203–8208. 10.1073/pnas.1806578115 30038023 PMC6094148

[B37] Dolphin A. C. (2018). Voltage-gated calcium channels: their discovery, function and importance as drug targets. Brain Neurosci. Adv. 2, 1–8. 10.1177/2398212818794805 30320224 PMC6179141

[B38] Dou Y. Macdonald L. C. Wu Y. Fedida D. (2017). The fast component of hERG gating charge: an interaction between D411 in the S1 and S4 residues. Biophys. J. 113 (9), 1979–1991. 10.1016/j.bpj.2017.09.004 29117522 PMC5685676

[B39] Doyle D. A. Morais Cabral J. Pfuetzner R. A. Kuo A. Gulbis J. M. Cohen S. L. (1998). The structure of the potassium channel: molecular basis of K+ conduction and selectivity. Science 280 (5360), 69–77. 10.1126/science.280.5360.69 9525859

[B40] Dudev T. Musset B. Morgan D. Cherny V. V. Smith S. M. Mazmanian K. (2015). Selectivity mechanism of the voltage-gated proton channel, HV1. Sci. Rep. 5, 10320. 10.1038/srpe10320 25955978 PMC4429351

[B41] Elinder F. Nilsson J. Arhem P. (2007). On the opening of voltage-gated ion channels. Physiol. Behav. 92 (1-2), 1–7. 10.1016/j.physbeh.2007.05.058 17585963

[B42] Fedida D. Hesketh J. C. (2001). Gating of voltage-gated potassium channels. Progr. Biophys. Mol. Biol. 75 (3), 165–199. 10.1016/S0079-6107(01)00006-2 11376798

[B43] Francis D. G. Rybalchenko V. Struyk A. Cannon S. C. (2011). Leaky sodium channels from voltage sensor mutations in periodic paralysis, but not paramyotonia. Neurol 76 (19), 1635–1641. 10.1212/WNL.0b013e318219fb57 PMC3100087 21490317

[B44] Freites J. A. Tobias D. J. (2015). Voltage-sensing in membranes: from macroscopic currents to molecular motions. J. Membr. Biol. 248 (3), 419–430. 10.1007/s00232-015-0805-x 25972106 PMC4490089

[B45] Freites J. A. Tobias D. J. White S. H. (2006). A voltage-sensor water pore. Biophys. J. 91 (11), L90–L92. 10.1529/biophysj.106.096065 17012321 PMC1635690

[B46] Fujiwara T. Sugawara T. Mazaki-Miyazaki E. Takahashi Y. Fukushima K. Watanabe M. (2003). Mutations of sodium channel a subunit type I (SCN1A) in intractable childhood epilepsies with frequent generalized tonic-clonic seizures. Brain 126, 531–546. 10.1093/brain/awg053 12566275

[B47] Gamal El-Din T. M. Martinez G. Q. Payandeh J. Scheuer T. Catterall W. A. (2013). A gating charge interaction required for late slow inactivation of the bacterial sodium channel Na_V_Ab. J. Gen. Physiol. 142 (3), 181–190. 10.1085/jgp.201311012 23980192 PMC3753604

[B48] Gandhi C. S. Isacoff E. Y. (2002). Molecular models of voltage sensing. J. Gen. Physiol. 120 (4), 455. 10.1085/jgp.20028678 12356848 PMC2229531

[B49] Ganetzky B. (2000). Genetic analysis of ion channel dysfunction in Drosophila. Kidney Int. 57 (3), 766–771. 10.1016/j.1523-1755.2000.00913.x 10720927

[B50] Garcia-Elias A. Benito B. (2018). Ion channel disorders and sudden cardiac death. Int. J. Mol. Sci. 19 (3), E692. 10.3390/ijms19030692 29495624 PMC5877553

[B51] Gianti E. Delemotte L. Klein M. Carvnevale V. (2016). On the role of water density fluctuations in the inhibition of a proton channel. Proc. Natl. Acad. Sci. U. S. A. 113 (52), E8359–E8368. 10.1073/pna.1609964114 27956641 PMC5206518

[B52] Glaudemans B. van der Wijst J. Scola R. H. Lorenzoni P. J. Heister A. van der Kemp A. W. (2009). A missense mutation in the Kv1.1 voltage-gated potassium channel-encoding gene KCNA1 is linked to human autosomal dominant hypomagnesemia. J. Clin. Invest. 119 (4), 936–942. 10.1172/JCI36948 19307729 PMC2662556

[B53] Goldschen-Ohm M. P. Capes D. L. Oelstrom K. M. Chanda B. (2013). Multiple pore conformations driven by asynchronous movements of voltage sensors in a eukaryotic sodium channel. Nat. Commun. 4, 1350. 10.1038/ncomms2356 23322038 PMC3562458

[B54] Gosselin-Badaroudine P. Delemotte L. Moreau A. Klein M. L. Chahine M. (2012). Gating pore currents and the resting state of Na_V_1.4 voltage sensor domains. Proc. Natl. Acad. Sci. U. S. A. 109 (47), 19250–19255. 10.1073/pnas.1217990109 23134726 PMC3511134

[B55] Greenblatt R. E. Blatt Y. Montal M. (1985). The structure of the voltage-sensitive sodium channel: inferences from computer-aided analysis of the Electrophorus electricus channel primary structure. FEBS 193 (2), 125–134. 10.1016/0014-5793(85)80136-8 2415395

[B56] Groenewegen W. A. Firouzi M. Bezzina C. R. Vliex S. van Langen I. M. Sandkuijl L. (2003). A cardiac sodium channel mutation cosegregates with a rare connexin40 genotype in familial atrial standstill. Circ. Res. 92 (1), 14–22. 10.1161/01.RES.0000050585.07097.D7 12522116

[B57] Groome J. R. Winston V. (2013). S1-S3 counter charges in the voltage sensor module of a mammalian sodium channel regulate fast inactivation. J. Gen. Physiol. 141 (5), 601–618. 10.1085/jgp.201210935 23589580 PMC3639575

[B58] Groome J. R. Moreau A. Delemotte L. (2018). “Gating pore currents in sodium channels,” in Handbook of Experimental Pharmacology, vol. 246 . Ed. Chahine M. Voltage-gated sodium channels: structure, function and channelopathies (Switzerland: Springer International Publishing AG), 371–399. 10.1007/164_2017_54 28965172

[B59] Groome J. R. Bayless-Edwards L. Wheeler A. Camp R. (2019). Domain I countercharges limit slow inactivation in hNa_V_1.4 channels. Biophys. J. 116 (Supplement Issue3), 390a. 10.1016/j.bpj.2018.11.2113

[B60] Groome J. R. (2014). “The voltage sensor module in sodium channels,” in Handbook of Experimental Pharmacology, vol. 221 . Ed. Ruben P. Voltage gated sodium channels (Berlin, Heidelberg: Springer International Publishing AG), 7–31. 10.1007/978-3.642-41588-3_2 24737230

[B61] Guy H. R. Seetharamulu P. (1986). Molecular model of the action potential sodium channel. Proc. Natl. Acad. Sci. U. S. A. 83, 508–512. 10.1073/pnas.83.2.508 2417247 PMC322889

[B62] Harkin L. A. McMahon J. M. Iona X. Dibbens L. Pelekanos J. T. Zuberi S. M. (2007). The spectrum of SNC1A-related infantile epileptic encephalopathies. Brain 130 (3), 843–852. 10.1093/brain/awm002 17347258

[B63] Hayano M. Makiyama T. Kamakura T. Watanabe H. Sasaki K. Funakoshi S. (2017). Development of a patient-derived induced pluripotent stem cell model for the investigation of SCN5A-D1275N-related cardiac sodium channelopathy. Circ. J. 81 (12), 1783–1791. 10.1253/circj.CJ-17-0064 28637969

[B64] Henrion U. Renhorn J. Borjesson S. I. Nelson E. M. Schwaiger C. S. Bjelkmar P. (2012). Tracking a complete voltage-sensor cycle with metal-ion bridges. Proc. Natl. Acad. Sci. U. S. A. 109 (22), 8552–8557. 10.1073/pnas.1116938109 22538811 PMC3365220

[B65] Hodgkin A. L. Huxley A. F. (1952). A quantitative description of membrane current and its application to conduction and excitation in nerve. J. Physiol. 117, 500–544. 10.1113/jphysiol.1952.sp004764 12991237 PMC1392413

[B66] Horn R. (2002). Coupled movements in voltage-gated ion channels. J. Gen. Physiol. 120 (4), 449–453. 10.1085/jgp.20028658 12356847 PMC2229539

[B67] Howard R. J. Carnevale V. Delemotte L. Hellmich U. A. Rothberg B. S. (2018). Permeating disciplines: overcoming barriers between molecular simulations and classical structure-function approaches in biological transport. Biochim. Biophys. Acta Biomembr. 1860 (4), 927–942. 10.1016/j.bbamem.2017.12.013 29258839 PMC6317864

[B68] Huang W. Liu M. Yan S. F. Yan N. (2017). Structure-based assessment of disease-related mutations in human voltage-gated sodium channels. Protein Cell 8 (6), 401–438. 10.1007/s13238-017-0372-z 28150151 PMC5445024

[B69] Huttner I. G. Trivedi G. Jacoby A. Mann S. A. Vandenberg J. I. Fatkin D. (2013). A transgenic zebrafish model of a human cardiac sodium channel mutation exhibits bradycardia, conduction-system abnormalities and early death. J. Mol. Cell. Cardiol. 61, 123–132. 10.1016/j.yjmcc.2013.06.005 23791817

[B70] Jensen M. Ø. Jojini V. Borhani D. W. Leffler A. E. Dror R. O. Shaw D. E. (2012). Mechanism of voltage gating in potassium channels. Science 336 (6078), 229–233. 10.1126/science.1216533 22499946

[B71] Jiang Y. Lee A. Chen J. Ruta V. Cadene M. Chait B. T. (2003). X-ray structure of a voltage-gated K+ channel. Nature 423 (6935), 33–41. 10.1038/nature01580 12721618

[B72] Jiang D. Gamal El-Din T. M. Ing C. Lu P. Pomes R. Zheng N. (2018). Structural basis for gating pore current in periodic paralysis. Nature 557 (7706), 590–594. 10.1038/s41586-018-0120-4 29769724 PMC6708612

[B73] Jogini V. Roux B. (2007). Dynamics of the Kv1.2 voltage-gated potassium channel in a membrane environment. Biophys. J. 93 (9), 3070–3082. 10.1529/biophysj.107.112540 17704179 PMC2025645

[B74] Jongbloed R. Marcelis C. Velter C. Doevendans P. Geraedts J. Smeets H. (2002). DHPLC analysis of potassium ion channel genes in congenital long QT syndrome. Hum. Mutat. 20 (5), 382–391. 10.1002/humu.10131 12402336

[B75] Kapplinger J. D. Tester D. J. Salisbury B. A. Carr J. L. Harris-Kerr C. Pollevick G. D. (2009). Spectrum and prevalence of mutations from the first 2,500 consecutive unrelated patients referred for the FAMILION® long QT syndrome genetic test. Heart Rhythm. 6 (9), 1297–1303. 10.1016/j.hrthm.2009.05.021 19716085 PMC3049907

[B76] Kapplinger J. D. Tester D. J. Alders M. Benito B. Berthet M. Brugada J. (2010). An international compendium of mutations in the SCN5A-encoded cardiac sodium channel in patients referred for Brugada syndrome genetic testing. Heart Rhythm. 7 (1), 33–46. 10.1016/j.hrthm.2009.090.069 20129283 PMC2822446

[B77] Ke Q. Ye J. Tang S. Wang J. Luo B. Ji F. (2017). N1366S mutation of human skeletal muscle sodium channel causes paramyotonia congenita. J. Physiol. 595 (22), 6837–6850. 10.1113/JP274877 28940424 PMC5685822

[B78] Kearney J. A. Wiste A. K. Stephani U. Trudeau M. S. Siegel A. RamachandranNair R. (2006). Recurrent *de novo* mutations of SCN1A in severe myoclonic epilepsy of infancy. Ped. Neurol. 34 (2), 116–120. 10.1016/j.pediatrneurol.2005.07.009 16458823

[B79] Khalili-Araghi F. Jogini V. Yarov-Yarovoy V. Tajkhorshid E. Roux B. Schulten K. (2010). Calculation of the gating charge for K_V_1.2 voltage-activated potassium channel. Biophys. J. 98 (10), 2189–2198. 10.1016/bpj.2010.02.056 20483327 PMC2872222

[B80] Kodera H. Kato M. Nord A. S. Walsh T. Lee M. Yamanaka G. (2013). Targeted capture and sequencing for detection of mutations causing early onset epileptic encephalopathy. Epilepsia 54 (7), 1262–1269. 10.1111/epi.12203 23662938

[B81] Kuang Q. Purhonen P. Hebert H. (2015). Structure of potassium channels. Cell. Mol. Life Sci. 72 (19), 3677–3693. 10.1007/s00018-015-1948-5 26070303 PMC4565861

[B82] Kubota T. Lacroix J. J. Bezanilla F. Correa A. M. (2014). Probing α-3_10_ transitions in a voltage-sensing S4 helix. Biophys. J. 107 (5), 1117–1128. 10.1016/j.bpj.2014.07.042 25185547 PMC4156671

[B83] Lacroix J. J. Hyde H. C. Campos F. V. Bezanilla F. (2014). Moving gating charges through the gating pore in a Kv channel voltage sensor. Proc. Natl. Acad. Sci. U. S. A. 111 (19), E1950–E1959. 10.1073/pnas.1406161111 24782544 PMC4024920

[B84] Leonard R. J. Karschin A. Jayashree-Aiyar S. Davidson N. Tanouye M. A. Thomas L. (1989). Expression of Drosophila Shaker potassium channels in mammalian cells infected with recombinant vaccina virus. Proc. Natl. Acad. Sci. U. S. A. 86 (19), 7629–7633. 10.1073/pnas.86.19.7629 2477844 PMC298120

[B85] Lin M.-C. A. Hsieh J.-Y. Mock A. F. Papazian D. M. (2011). R1 in the Shaker S4 occupies the gating charge transfer center in the resting state. J. Gen. Physiol. 138 (2), 155–163. 10.1085/jgp.201110642 21788609 PMC3149438

[B86] Liu J. Zhang M. Jiang M. Tseng G.-N. (2003). Negative charges in the transmembrane domains of the HERG K channel are involved in the activation- and deactivation-gating processes. J. Gen. Physiol. 121 (6), 599–614. 10.1085/jgp.200308788 12771194 PMC2217355

[B87] Long S. B. Campbell E. B. MacKinnon R. (2005a). Voltage sensor of K_V_1.2: structural basis of electromechanical coupling. Science 309 (5736), 903–908. 10.1126/science.1116270 16002579

[B88] Long S. B. Campbell E. B. MacKinnon R. (2005b). Crystal structure of a mammalian voltage-dependent Shaker family K+ channel. Science 309 (5736), 897–903. 10.1126/science.1116269 16002581

[B89] Long S. B. Tao X. Campbell E. B. MacKinnon R. (2007). Atomic structure of a voltage-dependent K+ channel in a lipid membrane-like environment. Nature 450 (7168), 376–382. 10.1038/nature06265 18004376

[B90] Marini C. Mei D. Temudo T. Ferrari A. R. Buti D. Dravet C. (2007). Idiopathic epilepsies with seizures precipitated by fever and SCN1A abnormalities. Epilepsia 48 (9), 1678–1685. 10.1111/j.1528-1167.2007.01122x 17561957

[B91] Matalon D. Goldberg E. Medne L. Marsh E. D. (2014). Confirming an expanded spectrum of SCN2A mutations: a case series. Epileptic Disord. 16 (1), 13–18. 10.1684/epd.2014.0641 24659627

[B92] McNair W. P. Ku L. Taylor M. R. G. Fain P. R. Dao D. Wolfel E. (2004). SCN5A mutation associated with dilated cardiomyopathy, conduction disorder, and arrhythmia. Circulation 110, 2163–2167. 10.1161/01.CIR.0000144458_58660.BB 15466643

[B93] Meregalli P. G. Tan H. L. Probst V. Koopmann T. T. Tanck M. W. Bhuiyan Z. A. (2009). Type of SCN5A-mutation determines clinical severity and degree of conduction slowing in loss-of-function sodium channelopathies. Heart Rhythm. 6 (3), 341–348. 10.1016/j.hrthm.2008.11.009 19251209

[B94] Monteleone S. Lieb A. Pinggera A. Negro G. Fuchs J. E. Hofer F. (2017). Mechanisms responsible for w-pore currents in Cav calcium channel voltage-sensing domains. Biophys. J. 113 (7), 1485–1495. 10.1016/j.bpj.2017.08.010 28978442 PMC5627182

[B95] Musset B. Smith S. M. E. Rajan S. Morgan D. Cherny V. V. DeCoursey T. E. (2012). Aspartate is the selectivity filter of the human voltage gated proton channel. Nature 480 (7376), 273–277. 10.1038/nature10557 PMC3237871 22020278

[B96] Nakamura K. Kato M. Osaka H. Yamashita S. Nakagawa E. Haginoya K. (2013). Clinical spectrum of SCN2A mutations expanding to Ohtahara syndrome. Neurol 81 (11), 992–998. 10.1212/WNL.0b013e3182a43e57 23935176

[B97] Napolitano C. Priori S. G. Schwartz P. J. Bloise R. Ronchetti E. Nastoli J. (2005). Genetic testing in the long QT syndrome: development and validation of an efficient approach to genotyping in clinical practice. JAMA 294 (23), 2975–2980. 10.1001/jama.294.23.2975 16414944

[B98] Nguyen T. P. Wang D. W. Rhodes T. H. George A. L. Jr. (2008). Divergent biophysical defects caused by mutant sodium channels in dilated cardiomyopathy with arrhythmia. Circ. Res. 102 (3), 364–371. 10.1161/CIRCRESAHA.107.164673 18048769

[B99] Nishizawa M. Nishizawa K. (2008). Molecular dynamics simulations of Kv channel voltage sensor helix in a lipid membrane with applied electric field. Biophys. J. 95 (4), L1729–L1744. 10.1529/biophysj.108.130658 PMC2483744 18487312

[B100] Noda M. Shimizu S. Tanabe T. Takai T. Kayano T. Ikeda T. (1984). Primary structure of Electrophorus electricus sodium channel deduced from cDNA sequence. Nature 312 (5990), 121–127. 10.1038/312121a0 6209577

[B101] Noda M. Ikeda T. Suzuki H. Takeshima H. Takahashi T. Kuno M. (1986). Expression of functional sodium channels from cloned DNA. Nature 322 (6082), 826–828. 10.1038/322826a0 2427955

[B102] Ogiwara I. Ito K. Sawaishi Y. Osaka H. Mazaki E. Inoue I. (2009). De novo mutations of voltage-gated sodium channel alphaII gene SCN2A in intractable epilepsies. Neurol 73 (13), 1046–1053. 10.1212/WNL.0b013e3181b0cebc PMC2754324 19786696

[B103] Olson T. M. Michels W. Ballew J. D. Reyna S. P. Karst M. L. Herron K. J. (2005). Sodium channel mutations and susceptibility to heart failure and atrial fibrillation. JAMA 293 (4), 447–454. 10.1001/jama.293.4.447 15671429 PMC2039897

[B104] Paldi T. Gurevitz M. (2010). Coupling between residues on S4 and S1 defines the voltage-sensor resting conformation in NaChBac. Biophys. J. 99 (2), 456–463. 10.1016/bpj.2010.04.053 20643063 PMC2905116

[B105] Palovcak E. Delemotte L. Klein M. L. Carnevale V. (2014). Evolutionary imprint of activation: the design principles of VSDs. J. Gen. Physiol. 143 (2), 145–156. 10.1085/jgp.201311103 24470486 PMC4001776

[B106] Pan X. Li Z. Zhou Q. Shen H. Wu K. Huang X. (2018). Structure of the human voltage-gated sodium channel Na_V_1.4 in complex with beta1. Science 362 (6412), eaau2486. 10.1126/science.aau2486 30190309

[B107] Pantazis A. Savalli N. Sigg D. Neely A. Olcese R. (2014). Functional heterogeneity of the four voltage sensors of a human L-type calcium channel. Proc. Natl. Acad. Sci. U. S. A. 111 (51), 18381–18386. 10.1073/pnas.1411127112 25489110 PMC4280600

[B108] Papazian D. M. Schwarz T. L. Tempel B. L. Jan Y. N. Jan L. Y. (1987). Cloning of genomic and complementary DNA from Shaker, a putative potassium gene from Drosophila. Science 237 (4816), 749–753. 10.1126/science.2441470 2441470

[B109] Papazian D. M. Schwarz T. L. Tempel B. L. Timpe L. C. Jan L. Y. (1988). Ion channels in Drosophila. Ann. Rev. Physiol. 50, 379–394. 10.1146/annurev.ph.50.030188.002115 2454072

[B110] Papazian D. M. Timpe L. C. Jan Y. N. Jan L. Y. (1991). Alteration of voltage-dependence of Shaker potassium channel by mutations in the S4 sequence. Nature 349 (6307), 305–310. 10.1038/349305a0 1846229

[B111] Papazian D. M. Shao X. M. Seoh S.-A. Mock A. F. Huang Y. Wainstock D. H. (1995). Electrostatic interactions of S4 voltage sensor in Shaker K+ channel. Neuron 14 (6), 1293–1301. 10.1016/0896-6273(95)90276-7 7605638

[B112] Papazian D. M. Silverman W. R. Lin M. C. Tiwari-Woodruff S. K. Tang C. Y. (2002). Structural organization of the voltage sensor in voltage-dependent K+ channels. Noavartis Found. Symp. 245, 178–190. 10.1002/0470868759.ch13 12027007

[B113] Parrini E. Marini C. Mei D. Galuppi A. Cellini E. Pucatti D. (2017). Diagnostic targeted resequencing in 349 patients with drug-resistant pediatric epilepsies identifies causative mutations in 30 different genes. Hum. Mutat. 38, 216–225. 10.1002/humu.23149 27864847

[B114] Pathak M. M. Yarov-Yarovoy V. Agarwal G. Roux B. Barth P. Kohout S. (2007). Closing in on the resting state of the shaker K+ channel. Neuron 56 (4), 124–140. 10.1016/j.neuron.2007.09.023 17920020

[B115] Payandeh J. Scheuer T. Zheng N. Catterall W. A. (2011). The crystal structure of a voltage-gated sodium channel. Nature 475 (7356), 353–358. 10.1038/nature10238 21743477 PMC3266868

[B116] Payandeh J. Gamal El-Din T. M. Scheuer T. Zheng N. Catterall W. A. (2012). Crystal structure of a voltage-gated sodium channel in two potentially inactivated states. Nature 486 (7401), 135–139. 10.1038/nature11077 22678296 PMC3552482

[B117] Perozo E. Papazian D. M. Stefani E. Bezanilla F. (1992). Gating currents in Shaker K+ channels. Implications for activation and inactivation models. Biophys. J. 62 (1), 160–168. 10.1016/S0006-3495(92)81802-7 1600094 PMC1260511

[B118] Perozo E. Santacruz-Toloza L. Stefani E. Bezanilla F. Papazian D. M. (1994). S4 mutations alter gating currents of Shaker K channels. Biophys. J. 66, 345–354. 10.1016/s0006-3495-(94)80783-0 8161688 PMC1275701

[B119] Pless S. A. Galpin J. D. Niciforovic A. P. Ahern C. A. (2011). Contributions of counter-charge in a potassium channel voltage-sensor domain. Nat. Chem. Biol. 7 (9), 617–623. 10.1038/nchembio.622 21785425 PMC4933587

[B120] Pless S. A. Elstone F. D. Niciforovic A. P. Galpin J. D. Yang R. Kurata H. T. (2014). Asymmetric functional contributions of acidic and aromatic side chains in sodium channel voltage-sensor domains. J. Gen. Physiol. 143 (5), 645–656. 10.1085/jgp.201311036 24778431 PMC4003186

[B121] Poulin H. Gosselin-Badaroudine P. Vicart S. Habbout K. Sternberg D. Giuliano S. (2018). Substitutions of the S4DIV R2 residue (R1451) in Na_V_1.4 lead to complex forms of paramyotonia congenita and periodic paralyses. Sci. Rep. 8, 2041. 10.1038/s41598-018-20468-0 29391559 PMC5794747

[B122] Ren D. Navarro B. Xu H. Yue L. Shi Q. Clapham D. E. (2001). A prokaryotic voltage-gated sodium channel. Science 294 (5550), 2372–2375. 10.1126/science.1065635 11743207

[B123] Schow E. V. Frietes J. A. Gogna K. White S. H. Tobias D. J. (2010). Down-state model of the voltage-sensing domain of a potassium channel. Biophys. J. 98 (112), 2857–2866. 10.1016/j.bpj.2010.03.031 20550898 PMC2884232

[B124] Schulteis C. T. Nagaya N. Papazian D. M. (1998). Subunit folding and assembly steps are interspersed during Shaker potassium channel biogenesis. J. Biol. Chem. 273 (40), 26210–26217. 10.1074/jbc.273.40.26210 9748304

[B125] Schwaiger C. S. Bjelkmar P. Hess B. Lindahl E. (2011). 3_10_-helix conformation facilitates the transition of a voltage sensor S4 segment toward the down state. Biophys. J. 100 (6), 1446–1454. 10.1016/j.bpj.2011.02.003 21402026 PMC3059565

[B126] Schwaiger C. S. Liin S. I. Elinder F. Lindahl E. (2013). The conserved phenylalanine in the K+ channel voltage-sensor domain creates a barrier with unidirectional effects. Biophys. J. 104 (1), 75–84. 10.1016/j.bpj.2012.11.3827 23332060 PMC3540256

[B127] Seoh S.-A. Sigg D. Papazian D. M. Bezanilla F. (1996). Voltage-sensing residues in the S2 and S4 segments of the Shaker K+ channel. Neuron 16, 1159–1167. 10.1016/s0896-6273(00)80142-7 8663992

[B128] Sercinoglu O. Ozbek P. (2018). gRINN: a tool for calculation of residue interaction energies and protein energy network analysis of molecular dynamics simulations. Nucleic Acids Res. 46 (W1), W554–W526. 10.1093/nar/gky381 29800260 PMC6030995

[B129] Shen H. Zhou Q. Pan X. Li Z. Wu J. Yan N. (2017). Structure of a eukaryotic voltage-gated sodium channel at near-atomic resolution. Science 355 (6328), eaa14326. 10.1126/scienceaa14326 28183995

[B130] Silverman W. R. Roux B. Papazian D. M. (2003). Structural basis of two-stage voltage dependent activation in K+ channels. Proc. Natl. Acad. Sci. U. S. A. 100 (5), 2935–2940. 10.1073/pnas.0636603100 12606713 PMC151444

[B131] Smits J. P. Eckardt L. Probst V. Bezzina C. R. Schott J. J. Remme C. A. (2002). Genotype-phenotype relationship in Brugada syndrome: electrocardiographic features differentiate SCN5A-related patients from non-SCN5A-related patients. J. Am. Coll. Cardiol. 40 (2), 350–356. 10.1016/s0735-1097(02)01962-9 12106943

[B132] Smits J. P. Koopmann T. T. Wilders R. Veldkamp M. W. Opthof T. Bhuiyan Z. A. (2005). A mutation in the human cardiac sodium channel E161K contributes to sick sinus syndrome, conduction disease, and Brugada syndrome in two families. J. Mol. Cell. Cardiol. 38 (6), 969–981. 10.1016/j.yjmcc.2005.02.024 15910881

[B133] Splawski I. Shen J. Timothy K. W. Lehmann M. H. Priori S. Robinson J. L. (2000). Spectrum of mutations in long-QT syndrome genes KVLQT1, HERG, SCN5A, KCNE1, and KCNE2. Circ 102, 1178–1185. 10.1161/01.cir.102.10.1178 10973849

[B134] Starace D. M. Bezanilla F. (2004). A proton pore in a potassium channel voltage sensor reveals a focused electric field. Nature 427 (6974), 548–553. 10.1038/nature02270 14765197

[B135] Stuhmer W. Stocker M. Sakmann B. Seeburg P. Baumann A. Grupe A. (1988). Potassium channels expressed from rat brain DNA have delayed rectifier properties. FEBS Lettr. 242 (1), 199–206. 10.1016/0014-5793(88)8105-9 2462513

[B136] Stuhmer W. Conti F. Suzuki H. Wang X. D. Noda M. Yahagi N. (1989). Structural parts involved in the activation and inactivation of the sodium channel. Nature 339 (6226), 597–603. 10.1038/339597a0 2543931

[B137] Sula A. Booker J. Ng L. C. T. Naylor C. E. DeCaen P. G. Wallace B. A. (2017). The complete structure of an activated open sodium channel. Nat. Commun. 8, 14205. 10.1038/ncomms14205 28205548 PMC5316852

[B138] Tao X. Lee A. Limapichat W. Dougherty D. A. MacKinnon R. (2010). A gating charge transfer center in voltage sensors. Science 328 (5974), 67–73. 10.1126/science.1185954 20360102 PMC2869078

[B139] Tester D. J. Will M. L. Haglund C. M. Ackermann M. J. (2005). Compendium of cardiac sodium channel mutations in 541 consecutive unrelated patients referred for long QT syndrome genetic testing. Heart Rhythm. 2 (5), 507–517. 10.1016/j.hrthm.2005.01.020 15840476

[B140] Timpe L. C. Schwarz T. L. Tempel B. L. Papazian D. M. Jan Y. N. Jan L. Y. (1988). Expression of functional potassium channels from Shaker cDNA in Xenopus oocytes. Nature 331 (6152), 143–145. 10.1038/331143a0 2448636

[B141] Tiwari-Woodruff S. K. Schulteis C. T. Mock A. F. Papazian D. M. (1997). Electrostatic interactions between transmembrane segments mediate folding of Shaker K+ channel subunits. Biophys. J. 72 (4), 1489–1500. 10.1016/S0006-3495(97)78797-6 9083655 PMC1184345

[B142] Tiwari-Woodruff S. K. Lin M.-A. Schulteis C. T. Papazian D. M. (2000). Voltage-dependent structural interactions in the Shaker K+ channel. J. Gen. Physiol. 115 (2), 123–138. 10.1085/jgp.115.2.123 10653892 PMC2217201

[B143] Tombola F. Pathak M. M. Isacoff E. Y. (2005). Voltage-sensing arginines in a potassium channel permeate and occlude cation-selective pores. Neuron 45 (3), 379–388. 10.1016/j.neuron.2004.12.047 15694325

[B144] Tombola F. Pathak M. M. Gorostiza P. Isacoff E. Y. (2007). The twisted ion-permeation pathway of a resting voltage-sensing domain. Nature 445 (7127), 546–549. 10.1038/nature05396 17187057

[B145] Treptow W. Tarek M. (2006). Environment of the gating charges in the Kv1.2 shaker potassium channel. Biophys. J. 90 (9), L64–L66. 10.1529/biophysj.106.080754 16533847 PMC1432113

[B146] Treptow W. Tarek M. Klein M. L. (2009). Initial response of the potassium channel voltage sensor to a transmembrane potential. J. Am. Chem. Soc 131 (6), 2107–2109. 10.1021/ja807330g 19175309 PMC2668160

[B147] Tuluc P. Benedetti B. Coste de Bagneaux P. Grabner M. Flucher B. E. (2016a). Two distinct voltage-sensing domains control voltage-sensitivity and kinetics of current activation in Cav1.1 calcium channels. J. Gen. Physiol. 147 (6), 437–439. 10.1085/jgp.201511568 27185857 PMC4886277

[B148] Tuluc P. Yarov-Yarovoy V. Benedetti B. Flucher B. E. (2016b). Molecular interactions in the voltage sensor controlling gating properties of Ca_V_ calcium channels. Structure 24 (2), 262–271. 10.1016/j.str.2015.11.011 PMC7360434 26749449

[B149] van Keulen S. C. Gianti E. Carnevale V. Klein M. L. Rothlisberger U. Delemotte L. (2017). Does proton conduction in the voltage-gated H+ channel hHv1 involve Grotthuss-like hopping *via* acidic residues? J. Phys. Chem. B 121, 3340–3351. 10.1021/acs.jpcb.6b08339 27801578 PMC6310143

[B150] Vargas E. Bezanilla F. Roux B. (2011). In search of a consensus model of the resting state of a voltage-sensing domain. Neuron 72 (5), 713–720. 10.1016/j.neuron.2011.09.024 22153369 PMC3268064

[B151] Vargas E. Yarov-Yarovoy V. Khalili-Araghi F. Catterall W. A. Klein M. L. Tarek M. (2012). An emerging consensus on voltage-dependent gating from computational modeling and molecular dynamics simulations. J. Gen. Physiol. 140 (6), 587–594. 10.1085/jgp.201210873 23183694 PMC3514734

[B152] Villalba-Galea C. A. Sandtner W. Starace D. M. Bezanilla F. (2008). S4-based voltage sensors have three major conformations. Proc. Natl. Acad. Sci. U. S. A. 105 (46), 17600–17607. 10.1073/pnas.0807387105 18818307 PMC2584729

[B153] Wang D. W. Viswanathan P. C. Balser J. R. George A. L. Jr. Benson D. W. (2002). Clinical, genetic, and biophysical characterization of SCN5A mutations associated with atrioventricular conduction block. Circ 105 (3), 341–346. 10.1161/hc0302.102592 11804990

[B154] Wang J.-W. Shi X.-Y. Kurahashi H. Hwang S.-K. Ishii A. Higurashi N. (2012). Prevalence of SCN1A mutations in children with suspected Dravet syndrome and intractable childhood epilepsy. Epilepsy Res. 102 (3), 195–200. 10.1016/j.eplepsyres.2012.06.006 23195492

[B155] Watanabe H. Yang T. Stroud D. M. Lowe J. S. Harris L. Atack T. C. (2011). Striking *in vivo* phenotype of a disease-associated human SCN5A mutation producing minimal changes *in vitro* . Circ 124 (9), 1001–1011. 10.1161/CIRCULATIONAHA.110.987248 PMC3297976 21824921

[B156] Wisedchaisri G. Tonggu L. McCord E. Gamal El-Din T. M. Wang L. Zheng N. (2019). Resting-state structure and gating mechanism of a voltage-gated sodium channel. Cell 178 (4), 993–1003. 10.1016/j.cell.2019.06.031 31353218 PMC6688928

[B157] Wong V. C. Fung C. W. Kwong A. K. (2015). SCN2A mutation in a Chinese boy with infantile spasm – response to modified Atkins diet. Brain Dev. 37 (7), 729–732. 10.1016/j.braindev.2014.10.008 25459969

[B158] Wood M. L. Schow E. V. Freites J. A. White S. H. Tombola F. Tobias D. J. (2012). Water wires in atomistic models of the Hv1 proton channel. Biochim. Biophys. Acta 1818 (2), 286–293. 10.1016/j.bbamem.2011.07.045 21843503 PMC3245885

[B159] Wu J. Yan Z. Li Z. Qian X. Lu S. Dong M. (2016). Structure of the voltage-gated calcium channel Ca(v)1.1 at 3.6 Å resolution. Nature 537 (7619), 191–196. 10.1038/nature19321 27580036

[B160] Yan Z. Zhou Q. Wang L. Wu J. Zhao Y. Huang G. (2017). Structure of the Na_V_1.4-beta1 complex from electric eel. Cell 170 (3), 470–482. 10.1016/j.cell.2017.06.039 28735751

[B161] Yarov-Yarovoy V. DeCaen P. G. (2019). The sodium channel voltage sensor slides to rest. Trends Pharmacol. Sci. 40 (10), 718–720. 10.1016/j.tips.2019.08.009 31495454 PMC7373202

[B162] Yarov-Yarovoy V. Baker D. Catterall W. A. (2006). Voltage sensor conformations in the open and closed states in ROSETTA structural models of K(+) channels. Proc. Natl. Acad. Sci. U. S. A. 103 (19), 7292–7297. 10.1073/pnas.0602350103 16648251 PMC1464335

[B163] Yarov-Yarovoy V. DeCaen P. G. Westenbroek R. E. Pan C. Y. Scheuer T. Baker D. (2012). Structural basis for gating charge movement in the voltage sensor of a sodium channel. Proc. Natl. Acad. Sci. U. S. A. 109 (2), E93–E102. 10.1073/pnas.1118434109 22160714 PMC3258622

[B164] Zagotta W. N. Toshi T. Aldrich R. W. (1989). Gating of single Shaker potassium channels in Drosophila muscle and in Xenopus oocytes injected with Shaker mRNA. Proc. Natl. Acad. Sci. U. S. A. 86 (18), 7243–7247. 10.1073/pnas.86.18.7243 2506548 PMC298033

[B165] Zaharieva I. T. Thor M. G. Oates E. C. van Karnebeek C. Hendson G. Blom E. (2016). Loss-of-function mutations in SCN4A cause severe foetal hypokinesia or ‘classical’ congenital myopathy. Brain 139 (3), 674–691. 10.1093/brain/awv352 26700687 PMC4766374

[B166] Zhang M. Liu J. Jiang M. Wu D.-M. Sonawane K. Guy H. R. (2005). Interactions between charged residues in the transmembrane segments of the voltage-sensing domain in the hERG channel. J. Mem. Biol. 207 (3), 169–181. 10.1007/s00232-005-0812-1 16550488

[B167] Zhang X. Ren W. DeCaen P. Yan C. Tao X. Tang. L. (2012). Crystal structure of an orthologue of the NaChBac voltage-gated sodium channel. Nature 486 (7401), 130–134. 10.1038/nature11054 22678295 PMC3979295

[B168] Zuberi S. M. Brunklaus A. Birch R. Reavey E. Duncan J. Forbes G. H. (2011). Genotype-phenotype associations in SCN1A-epilepsies. Neurol 76 (7), 594–600. 10.1212/WNL.0b013e31820c309b 21248271

